# Chlorido{*N*-[(diethyl­amino)­dimethyl­sil­yl]anilido-κ*N*}(*N*,*N*,*N*′,*N*′-tetra­methyl­ethane-1,2-diamine-κ^2^ *N*,*N*′)cobalt(II)

**DOI:** 10.1107/S1600536811002959

**Published:** 2011-01-29

**Authors:** Sheng-Di Bai, Min Hu

**Affiliations:** a Institute of Applied Chemistry Shanxi University, Taiyuan 030006, People’s Republic of China

## Abstract

In the title cobalt(II) compound, [Co(C_12_H_21_N_2_Si)Cl(C_6_H_16_N_2_)], the ethane-1,2-diamine donor mol­ecule coordin­ates the metal atom in an *N*,*N*′-chelating mode, with Co—N distances of 2.136 (2) and 2.140 (3) Å. An anilide ligand connects to the Co^II^ atom with a σ–bond, the Co—N_anilide_ distance being 1.931 (2) Å. The four-coordinate Co^II^ atom demonstrates a slightly distorted tetra­hedral geometry.

## Related literature

For reviews of related metal amides, see: Holm *et al.* (1996 ▶); Kempe (2000 ▶). For the catalytic applications of related *N*–silylated analido–group 4 metal compounds towards olefin polymerization, see: Gibson *et al.* (1998 ▶); Hill & Hitchcock (2002 ▶); Yuan *et al.* (2010 ▶). For related organometallic compounds with analogous analido ligands, see: Schumann *et al.* (2000 ▶); Chen (2008 ▶, 2009 ▶).
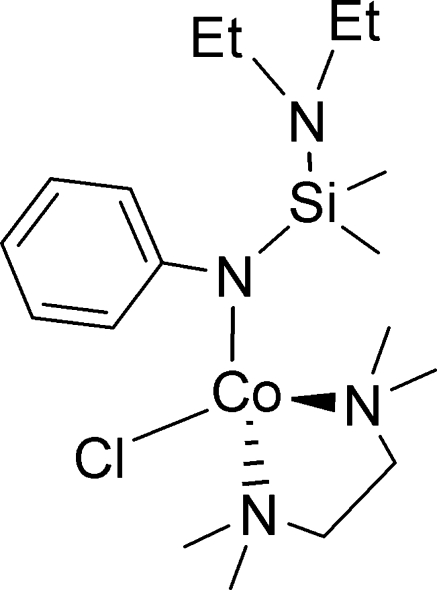


## Experimental

### 

#### Crystal data

[Co(C_12_H_21_N_2_Si)Cl(C_6_H_16_N_2_)]
*M* *_r_* = 431.99
Monoclinic, 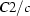

*a* = 20.711 (2) Å
*b* = 7.7110 (8) Å
*c* = 29.844 (3) Å
β = 99.009 (2)°
*V* = 4707.4 (8) Å^3^
*Z* = 8
Mo *K*α radiation
μ = 0.90 mm^−1^
*T* = 295 K
0.30 × 0.25 × 0.20 mm

#### Data collection

Bruker SMART CCD diffractometer
Absorption correction: multi-scan (*SADABS*; Sheldrick, 1996 ▶) *T*_min_ = 0.774, *T*_max_ = 0.840
13181 measured reflections
4630 independent reflections
3530 reflections with *I* > 2σ(*I*)
*R*_int_ = 0.035

#### Refinement

*R*[*F* ^2^ > 2σ(*F* ^2^)] = 0.048
*wR*(*F* ^2^) = 0.134
*S* = 1.05
4630 reflections
226 parameters
H-atom parameters constrained
Δρ_max_ = 0.58 e Å^−3^
Δρ_min_ = −0.35 e Å^−3^

### 

Data collection: *SMART* (Bruker, 2000 ▶); cell refinement: *SAINT* (Bruker, 2000 ▶); data reduction: *SAINT*; program(s) used to solve structure: *SHELXS97* (Sheldrick, 2008 ▶); program(s) used to refine structure: *SHELXL97* (Sheldrick, 2008 ▶); molecular graphics: *SHELXTL* (Sheldrick, 2008 ▶); software used to prepare material for publication: *SHELXL97*.

## Supplementary Material

Crystal structure: contains datablocks I, global. DOI: 10.1107/S1600536811002959/rk2258sup1.cif

Structure factors: contains datablocks I. DOI: 10.1107/S1600536811002959/rk2258Isup2.hkl

Additional supplementary materials:  crystallographic information; 3D view; checkCIF report

## Figures and Tables

**Fig. 1.**
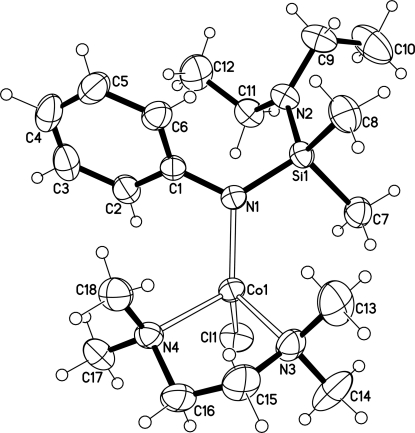
The molecular structure, showing the atom–numbering scheme. Displacement ellipsoids are drawn at the 30% probability level. H atoms are presented as a small spheres of arbitrary radius.

## supplementary crystallographic information

### Comment

Metal amides were important substitutes for cyclopentadienyl derivatives. They were found having valuable applications in various industrial and biological processes (Holm *et al.*, 1996; Kempe, 2000). Group 4 metal amides supported with the *N*–silylated anilido ligands were active catalysts for olefin polymerization (Gibson *et al.*, 1998; Hill & Hitchcock, 2002). Moreover, a class of monoionic *N*–silylated anilido–ligands bearing a pendant amino–group were paid much attentions. It was presumed that the empty *d*–orbitals on silicon would interact with the lone–pair electrons on the *p*–orbital of nitrogen center through *d—p**π* interaction throughout the N—Si—N motif. Analogous compounds with different metals including Zn (Schumann *et al.*, 2000), Zr (Chen, 2009) and Fe (Chen, 2008) have been synthesized. A group of zirconium amides with the similar ligand were reported showing good performance in ethylene polymerization (Yuan *et al.*, 2010). Here, the synthesis and crystal structure of a new cobalt(II) anilido–complex will be described.

The title compound was prepared by a one–pot reaction of *n*–*Bu*Li, *N*–[(diethylamino)dimethylsilyl]aniline, 1,2–bis(dimethylamino)ethane (*tmeda*) and CoCl_2_. The suitable for X–ray investigation single–crystal of the title compound was obtained by recrystallization in toluene. Its molecular structure is shown in Fig. 1. In the monomeric molecular structure of title compound, the metal Co center is coordinated by a chlorine atom, a chelating *tmeda* molecule and the anilido–ligand. The neutral donor molecule coordinates metal center in *N*,*N'*–chelating mode. Though the anilido–ligand has a pendant amino group, exhibting an N—Si—N chelating moiety, it connects Co(II) only with a σ–bond, Co—N_anilido_ being 1.931 (2)Å. It suggests the less affinity between the pendant amino–group and the metal center in comparing with *tmeda*. The angle of N1—Si1—N2 is 110.18 (12)°. The four–coordinate Co atom demonstrates a slightly distorted tetrahedral geometry. In the cases of N1—Si1—N1 biting metal center, the angles were constrained to less than 100°.

### Experimental

A solution of *n*–*Bu*Li (1.6 *M*, 1.9 ml, 3.1 mmol) in hexane was slowly added into a mixture of *N*–[(diethylamino)dimethylsilyl]aniline (0.69 g, 3.1 mmol) and *tmeda* (0.36 g, 3.1 mmol) in *Et*_2_O (20 ml) at 273 K by syringe. The mixture was stirred at room temperature for two hours and then added to a stirring suspension of CoCl_2_ (0.41 g, 3.1 mmol) in *Et*_2_O (20 ml) at 273 K. The resulting mixture was stirred at room temperature for 8 h. Then all the volatiles were removed under vacuum. The residue was extracted with toluene (25 ml). The filtrate was concentrated to give the title compound as green crystals (yield 0.52 g, 39%). M.p.: 390–391 K. MS (EI, 70 eV): *m/z* 431 [*M*]^+^. Anal. Calc. for C_18_H_37_ClCoN_4_Si: C, 50.05; H, 8.63; N, 12.97%.Found: C, 49.20; H, 8.37; N, 12.59%.

### Refinement

The methyl H atoms were constrained to an ideal geometry, with C—H distances of 0.96Å and *U*_iso_(H) = 1.5*U*_eq_(C), but each group was allowed to rotate freely about its C—C, C—N and C—Si bonds. The methylene H atoms were constrained with C—H distances of 0.97Å and *U*_iso_(H) = 1.2*U*_eq_(C). The phenyl H atoms were placed in geometrically idealized positions and constrained to ride on their parent atoms, with C—H distances in the range 0.93Å and *U*_iso_(H) = 1.2*U*_eq_(C).

### Crystal data

**Table:**

[Co(C_12_H_21_N_2_Si)Cl(C_6_H_16_N_2_)]	*F*(000) = 1848
*M**_r_* = 431.99	*D*_x_ = 1.219 Mg m^−^^3^
Monoclinic, *C*2/*c*	Melting point = 390–391 K
Hall symbol: -C 2yc	Mo *K*α radiation, λ = 0.71073 Å
*a* = 20.711 (2) Å	Cell parameters from 2838 reflections
*b* = 7.7110 (8) Å	θ = 2.6–27.3°
*c* = 29.844 (3) Å	µ = 0.90 mm^−^^1^
β = 99.009 (2)°	*T* = 295 K
*V* = 4707.4 (8) Å^3^	Block, green
*Z* = 8	0.30 × 0.25 × 0.20 mm

### Data collection

**Table:**

Bruker SMART CCD diffractometer	4630 independent reflections
Radiation source: fine-focus sealed tube	3530 reflections with *I* > 2σ(*I*)
graphite	*R*_int_ = 0.035
φ and ω scans	θ_max_ = 26.0°, θ_min_ = 1.4°
Absorption correction: multi-scan (*SADABS*; Sheldrick, 1996)	*h* = −25→19
*T*_min_ = 0.774, *T*_max_ = 0.840	*k* = −9→9
13181 measured reflections	*l* = −33→36

### Refinement

**Table:**

Refinement on *F*^2^	Primary atom site location: structure-invariant direct methods
Least-squares matrix: full	Secondary atom site location: difference Fourier map
*R*[*F*^2^ > 2σ(*F*^2^)] = 0.048	Hydrogen site location: inferred from neighbouring sites
*wR*(*F*^2^) = 0.134	H-atom parameters constrained
*S* = 1.05	*w* = 1/[σ^2^(*F*_o_^2^) + (0.0786*P*)^2^ + 0.8817*P*] where *P* = (*F*_o_^2^ + 2*F*_c_^2^)/3
4630 reflections	(Δ/σ)_max_ = 0.002
226 parameters	Δρ_max_ = 0.58 e Å^−^^3^
0 restraints	Δρ_min_ = −0.35 e Å^−^^3^

### Special details

**Table:**

Geometry. All s.u.'s (except the s.u. in the dihedral angle between two l.s. planes) are estimated using the full covariance matrix. The cell s.u.'s are taken into account individually in the estimation of s.u.'s in distances, angles and torsion angles; correlations between s.u.'s in cell parameters are only used when they are defined by crystal symmetry. An approximate (isotropic) treatment of cell s.u.'s is used for estimating s.u.'s involving l.s. planes.
Refinement. Refinement of *F*^2^ against ALL reflections. The weighted *R*–factor *wR* and goodness of fit *S* are based on *F*^2^, conventional *R*–factors *R* are based on *F*, with *F* set to zero for negative *F*^2^. The threshold expression of *F*^2^ > σ(*F*^2^) is used only for calculating *R*–factors(gt) *etc*. and is not relevant to the choice of reflections for refinement. *R*–factors based on *F*^2^ are statistically about twice as large as those based on *F*, and *R*–factors based on ALL data will be even larger.

### Fractional atomic coordinates and isotropic or equivalent isotropic displacement parameters (Å^2^)

**Table:**

	*x*	*y*	*z*	*U*_iso_*/*U*_eq_	
Co1	0.409198 (18)	0.55218 (5)	0.588284 (12)	0.04797 (15)	
Si1	0.37709 (4)	0.69888 (11)	0.68265 (3)	0.0512 (2)	
Cl1	0.44296 (5)	0.76779 (12)	0.54620 (3)	0.0749 (3)	
N1	0.35134 (11)	0.6003 (3)	0.63160 (8)	0.0499 (6)	
N2	0.32211 (13)	0.8549 (4)	0.69276 (8)	0.0615 (7)	
N3	0.48921 (13)	0.3793 (4)	0.60883 (10)	0.0685 (7)	
N4	0.37541 (12)	0.3566 (3)	0.53954 (8)	0.0561 (6)	
C1	0.28473 (13)	0.5721 (4)	0.61545 (10)	0.0514 (7)	
C2	0.25669 (16)	0.6353 (5)	0.57293 (11)	0.0669 (9)	
H2A	0.2818	0.7003	0.5558	0.080*	
C3	0.1910 (2)	0.6013 (6)	0.55597 (15)	0.0862 (13)	
H3A	0.1733	0.6430	0.5275	0.103*	
C4	0.15266 (19)	0.5085 (6)	0.5803 (2)	0.0968 (16)	
H4A	0.1092	0.4859	0.5685	0.116*	
C5	0.17918 (19)	0.4492 (5)	0.62229 (17)	0.0860 (12)	
H5A	0.1532	0.3871	0.6394	0.103*	
C6	0.24433 (16)	0.4799 (5)	0.63991 (13)	0.0668 (9)	
H6A	0.2612	0.4380	0.6686	0.080*	
C7	0.45913 (16)	0.7966 (5)	0.67824 (12)	0.0706 (9)	
H7A	0.4551	0.8725	0.6525	0.106*	
H7B	0.4897	0.7058	0.6747	0.106*	
H7C	0.4745	0.8612	0.7053	0.106*	
C8	0.3881 (2)	0.5524 (5)	0.73357 (12)	0.0802 (11)	
H8A	0.3468	0.5024	0.7371	0.120*	
H8B	0.4048	0.6182	0.7602	0.120*	
H8C	0.4184	0.4617	0.7294	0.120*	
C9	0.3114 (2)	0.9125 (5)	0.73794 (13)	0.0838 (11)	
H9A	0.3172	0.8142	0.7585	0.101*	
H9B	0.2665	0.9515	0.7360	0.101*	
C10	0.3563 (3)	1.0566 (7)	0.75764 (17)	0.134 (2)	
H10A	0.3466	1.0873	0.7870	0.201*	
H10B	0.3501	1.1558	0.7380	0.201*	
H10C	0.4009	1.0183	0.7604	0.201*	
C11	0.29799 (18)	0.9767 (5)	0.65620 (12)	0.0687 (9)	
H11A	0.3144	0.9416	0.6289	0.082*	
H11B	0.3153	1.0912	0.6645	0.082*	
C12	0.2238 (2)	0.9869 (6)	0.64612 (17)	0.1019 (14)	
H12A	0.2110	1.0683	0.6220	0.153*	
H12B	0.2072	1.0242	0.6728	0.153*	
H12C	0.2063	0.8746	0.6372	0.153*	
C13	0.4960 (2)	0.3208 (6)	0.65667 (14)	0.1036 (15)	
H13A	0.5329	0.2443	0.6631	0.155*	
H13B	0.4571	0.2606	0.6614	0.155*	
H13C	0.5025	0.4196	0.6764	0.155*	
C14	0.55074 (19)	0.4609 (7)	0.6014 (2)	0.1199 (19)	
H14A	0.5864	0.3826	0.6106	0.180*	
H14B	0.5575	0.5656	0.6189	0.180*	
H14C	0.5486	0.4878	0.5698	0.180*	
C15	0.4741 (2)	0.2226 (5)	0.57985 (15)	0.0912 (13)	
H15A	0.5147	0.1658	0.5759	0.109*	
H15B	0.4488	0.1418	0.5950	0.109*	
C16	0.43702 (18)	0.2672 (5)	0.53474 (13)	0.0784 (11)	
H16A	0.4634	0.3417	0.5187	0.094*	
H16B	0.4273	0.1622	0.5171	0.094*	
C17	0.3459 (2)	0.4245 (5)	0.49423 (11)	0.0786 (10)	
H17A	0.3319	0.3292	0.4744	0.118*	
H17B	0.3779	0.4916	0.4817	0.118*	
H17C	0.3091	0.4964	0.4974	0.118*	
C18	0.32867 (18)	0.2356 (5)	0.55483 (13)	0.0760 (10)	
H18A	0.3153	0.1516	0.5315	0.114*	
H18B	0.2911	0.2986	0.5612	0.114*	
H18C	0.3490	0.1775	0.5818	0.114*	

### Atomic displacement parameters (Å^2^)

**Table:**

	*U*^11^	*U*^22^	*U*^33^	*U*^12^	*U*^13^	*U*^23^
Co1	0.0462 (2)	0.0454 (2)	0.0532 (2)	0.00471 (16)	0.01055 (16)	0.00056 (16)
Si1	0.0552 (5)	0.0527 (5)	0.0444 (4)	−0.0009 (4)	0.0040 (3)	0.0029 (3)
Cl1	0.0905 (6)	0.0578 (5)	0.0813 (6)	−0.0065 (4)	0.0282 (5)	0.0086 (4)
N1	0.0457 (12)	0.0546 (14)	0.0501 (13)	0.0050 (11)	0.0091 (10)	−0.0031 (11)
N2	0.0739 (17)	0.0605 (17)	0.0522 (14)	0.0004 (13)	0.0162 (13)	−0.0043 (12)
N3	0.0522 (15)	0.0673 (18)	0.0827 (19)	0.0155 (13)	0.0004 (14)	−0.0027 (15)
N4	0.0575 (15)	0.0519 (15)	0.0607 (14)	0.0012 (12)	0.0146 (12)	−0.0068 (12)
C1	0.0471 (15)	0.0502 (17)	0.0565 (16)	0.0123 (13)	0.0072 (13)	−0.0146 (13)
C2	0.066 (2)	0.070 (2)	0.0608 (18)	0.0201 (17)	0.0009 (16)	−0.0090 (16)
C3	0.076 (2)	0.082 (3)	0.089 (3)	0.035 (2)	−0.024 (2)	−0.032 (2)
C4	0.049 (2)	0.091 (3)	0.143 (4)	0.015 (2)	−0.006 (3)	−0.061 (3)
C5	0.058 (2)	0.083 (3)	0.120 (3)	−0.0094 (19)	0.024 (2)	−0.039 (3)
C6	0.0573 (18)	0.065 (2)	0.080 (2)	0.0011 (16)	0.0160 (17)	−0.0139 (17)
C7	0.066 (2)	0.073 (2)	0.071 (2)	−0.0095 (18)	0.0050 (17)	−0.0026 (18)
C8	0.100 (3)	0.075 (3)	0.061 (2)	0.001 (2)	−0.0007 (19)	0.0179 (18)
C9	0.110 (3)	0.079 (3)	0.069 (2)	0.004 (2)	0.033 (2)	−0.0084 (19)
C10	0.208 (7)	0.112 (4)	0.084 (3)	−0.037 (4)	0.032 (4)	−0.038 (3)
C11	0.082 (2)	0.057 (2)	0.067 (2)	0.0101 (17)	0.0106 (18)	−0.0003 (16)
C12	0.091 (3)	0.093 (3)	0.116 (3)	0.031 (3)	0.000 (3)	−0.007 (3)
C13	0.114 (3)	0.088 (3)	0.097 (3)	0.037 (3)	−0.019 (3)	0.013 (2)
C14	0.048 (2)	0.122 (4)	0.189 (6)	0.014 (2)	0.018 (3)	0.004 (4)
C15	0.079 (3)	0.069 (3)	0.123 (4)	0.032 (2)	0.009 (2)	−0.016 (2)
C16	0.073 (2)	0.075 (3)	0.091 (3)	0.0126 (19)	0.024 (2)	−0.021 (2)
C17	0.096 (3)	0.082 (3)	0.0575 (19)	0.002 (2)	0.0097 (18)	−0.0130 (18)
C18	0.085 (2)	0.060 (2)	0.086 (2)	−0.0138 (18)	0.022 (2)	−0.0144 (18)

### Geometric parameters (Å, °)

**Table:**

Co1—N1	1.931 (2)	C8—H8B	0.9600
Co1—N4	2.136 (2)	C8—H8C	0.9600
Co1—N3	2.140 (3)	C9—C10	1.508 (6)
Co1—Cl1	2.2595 (9)	C9—H9A	0.9700
Si1—N1	1.711 (2)	C9—H9B	0.9700
Si1—N2	1.715 (3)	C10—H10A	0.9600
Si1—C8	1.878 (3)	C10—H10B	0.9600
Si1—C7	1.882 (3)	C10—H10C	0.9600
N1—C1	1.405 (4)	C11—C12	1.521 (5)
N2—C11	1.467 (4)	C11—H11A	0.9700
N2—C9	1.469 (4)	C11—H11B	0.9700
N3—C14	1.469 (5)	C12—H12A	0.9600
N3—C13	1.483 (5)	C12—H12B	0.9600
N3—C15	1.491 (5)	C12—H12C	0.9600
N4—C18	1.468 (4)	C13—H13A	0.9600
N4—C16	1.477 (4)	C13—H13B	0.9600
N4—C17	1.489 (4)	C13—H13C	0.9600
C1—C6	1.389 (5)	C14—H14A	0.9600
C1—C2	1.398 (4)	C14—H14B	0.9600
C2—C3	1.399 (5)	C14—H14C	0.9600
C2—H2A	0.9300	C15—C16	1.482 (5)
C3—C4	1.360 (7)	C15—H15A	0.9700
C3—H3A	0.9300	C15—H15B	0.9700
C4—C5	1.367 (7)	C16—H16A	0.9700
C4—H4A	0.9300	C16—H16B	0.9700
C5—C6	1.390 (5)	C17—H17A	0.9600
C5—H5A	0.9300	C17—H17B	0.9600
C6—H6A	0.9300	C17—H17C	0.9600
C7—H7A	0.9600	C18—H18A	0.9600
C7—H7B	0.9600	C18—H18B	0.9600
C7—H7C	0.9600	C18—H18C	0.9600
C8—H8A	0.9600		
			
N1—Co1—N4	114.84 (10)	N2—C9—C10	114.1 (3)
N1—Co1—N3	117.50 (11)	N2—C9—H9A	108.7
N4—Co1—N3	84.94 (10)	C10—C9—H9A	108.7
N1—Co1—Cl1	120.61 (8)	N2—C9—H9B	108.7
N4—Co1—Cl1	103.76 (7)	C10—C9—H9B	108.7
N3—Co1—Cl1	108.92 (9)	H9A—C9—H9B	107.6
N1—Si1—N2	110.18 (12)	C9—C10—H10A	109.5
N1—Si1—C8	115.75 (16)	C9—C10—H10B	109.5
N2—Si1—C8	106.22 (16)	H10A—C10—H10B	109.5
N1—Si1—C7	105.93 (14)	C9—C10—H10C	109.5
N2—Si1—C7	111.30 (16)	H10A—C10—H10C	109.5
C8—Si1—C7	107.49 (18)	H10B—C10—H10C	109.5
C1—N1—Si1	121.77 (18)	N2—C11—C12	113.3 (3)
C1—N1—Co1	114.80 (18)	N2—C11—H11A	108.9
Si1—N1—Co1	122.79 (13)	C12—C11—H11A	108.9
C11—N2—C9	114.0 (3)	N2—C11—H11B	108.9
C11—N2—Si1	118.4 (2)	C12—C11—H11B	108.9
C9—N2—Si1	125.0 (3)	H11A—C11—H11B	107.7
C14—N3—C13	108.7 (4)	C11—C12—H12A	109.5
C14—N3—C15	111.6 (3)	C11—C12—H12B	109.5
C13—N3—C15	107.0 (3)	H12A—C12—H12B	109.5
C14—N3—Co1	110.0 (3)	C11—C12—H12C	109.5
C13—N3—Co1	114.7 (2)	H12A—C12—H12C	109.5
C15—N3—Co1	104.9 (2)	H12B—C12—H12C	109.5
C18—N4—C16	110.8 (3)	N3—C13—H13A	109.5
C18—N4—C17	108.0 (3)	N3—C13—H13B	109.5
C16—N4—C17	108.3 (3)	H13A—C13—H13B	109.5
C18—N4—Co1	113.51 (19)	N3—C13—H13C	109.5
C16—N4—Co1	101.5 (2)	H13A—C13—H13C	109.5
C17—N4—Co1	114.5 (2)	H13B—C13—H13C	109.5
C6—C1—C2	117.2 (3)	N3—C14—H14A	109.5
C6—C1—N1	122.6 (3)	N3—C14—H14B	109.5
C2—C1—N1	120.2 (3)	H14A—C14—H14B	109.5
C1—C2—C3	120.3 (4)	N3—C14—H14C	109.5
C1—C2—H2A	119.8	H14A—C14—H14C	109.5
C3—C2—H2A	119.8	H14B—C14—H14C	109.5
C4—C3—C2	121.4 (4)	C16—C15—N3	111.7 (3)
C4—C3—H3A	119.3	C16—C15—H15A	109.3
C2—C3—H3A	119.3	N3—C15—H15A	109.3
C3—C4—C5	118.7 (4)	C16—C15—H15B	109.3
C3—C4—H4A	120.6	N3—C15—H15B	109.3
C5—C4—H4A	120.6	H15A—C15—H15B	107.9
C4—C5—C6	121.1 (4)	N4—C16—C15	110.7 (3)
C4—C5—H5A	119.4	N4—C16—H16A	109.5
C6—C5—H5A	119.4	C15—C16—H16A	109.5
C1—C6—C5	121.2 (4)	N4—C16—H16B	109.5
C1—C6—H6A	119.4	C15—C16—H16B	109.5
C5—C6—H6A	119.4	H16A—C16—H16B	108.1
Si1—C7—H7A	109.5	N4—C17—H17A	109.5
Si1—C7—H7B	109.5	N4—C17—H17B	109.5
H7A—C7—H7B	109.5	H17A—C17—H17B	109.5
Si1—C7—H7C	109.5	N4—C17—H17C	109.5
H7A—C7—H7C	109.5	H17A—C17—H17C	109.5
H7B—C7—H7C	109.5	H17B—C17—H17C	109.5
Si1—C8—H8A	109.5	N4—C18—H18A	109.5
Si1—C8—H8B	109.5	N4—C18—H18B	109.5
H8A—C8—H8B	109.5	H18A—C18—H18B	109.5
Si1—C8—H8C	109.5	N4—C18—H18C	109.5
H8A—C8—H8C	109.5	H18A—C18—H18C	109.5
H8B—C8—H8C	109.5	H18B—C18—H18C	109.5
			
N2—Si1—N1—C1	−34.7 (3)	N1—Co1—N4—C16	−142.6 (2)
C8—Si1—N1—C1	85.8 (3)	N3—Co1—N4—C16	−24.6 (2)
C7—Si1—N1—C1	−155.2 (2)	Cl1—Co1—N4—C16	83.7 (2)
N2—Si1—N1—Co1	135.65 (16)	N1—Co1—N4—C17	101.0 (2)
C8—Si1—N1—Co1	−103.8 (2)	N3—Co1—N4—C17	−141.0 (2)
C7—Si1—N1—Co1	15.2 (2)	Cl1—Co1—N4—C17	−32.7 (2)
N4—Co1—N1—C1	−29.3 (2)	Si1—N1—C1—C6	−56.6 (4)
N3—Co1—N1—C1	−126.8 (2)	Co1—N1—C1—C6	132.4 (2)
Cl1—Co1—N1—C1	96.0 (2)	Si1—N1—C1—C2	124.4 (3)
N4—Co1—N1—Si1	159.74 (14)	Co1—N1—C1—C2	−46.6 (3)
N3—Co1—N1—Si1	62.2 (2)	C6—C1—C2—C3	−1.7 (4)
Cl1—Co1—N1—Si1	−74.92 (17)	N1—C1—C2—C3	177.3 (3)
N1—Si1—N2—C11	−46.8 (3)	C1—C2—C3—C4	0.8 (5)
C8—Si1—N2—C11	−172.9 (3)	C2—C3—C4—C5	0.5 (6)
C7—Si1—N2—C11	70.4 (3)	C3—C4—C5—C6	−1.0 (6)
N1—Si1—N2—C9	152.6 (3)	C2—C1—C6—C5	1.3 (5)
C8—Si1—N2—C9	26.5 (3)	N1—C1—C6—C5	−177.7 (3)
C7—Si1—N2—C9	−90.2 (3)	C4—C5—C6—C1	0.1 (5)
N1—Co1—N3—C14	−127.3 (3)	C11—N2—C9—C10	−74.0 (5)
N4—Co1—N3—C14	117.3 (3)	Si1—N2—C9—C10	87.3 (5)
Cl1—Co1—N3—C14	14.5 (3)	C9—N2—C11—C12	−69.9 (4)
N1—Co1—N3—C13	−4.4 (3)	Si1—N2—C11—C12	127.4 (3)
N4—Co1—N3—C13	−119.8 (3)	C14—N3—C15—C16	−87.8 (4)
Cl1—Co1—N3—C13	137.4 (3)	C13—N3—C15—C16	153.4 (3)
N1—Co1—N3—C15	112.6 (3)	Co1—N3—C15—C16	31.2 (4)
N4—Co1—N3—C15	−2.8 (3)	C18—N4—C16—C15	−71.2 (4)
Cl1—Co1—N3—C15	−105.6 (2)	C17—N4—C16—C15	170.5 (3)
N1—Co1—N4—C18	−23.7 (3)	Co1—N4—C16—C15	49.7 (4)
N3—Co1—N4—C18	94.3 (2)	N3—C15—C16—N4	−58.0 (5)
Cl1—Co1—N4—C18	−157.4 (2)		
